# Treatment of Acute Tacrolimus Toxicity with Phenytoin in Solid Organ Transplant Recipients

**DOI:** 10.1155/2013/375263

**Published:** 2013-06-13

**Authors:** Arin S. Jantz, Samir J. Patel, Wadi N. Suki, Richard J. Knight, Arvind Bhimaraj, A. Osama Gaber

**Affiliations:** ^1^Department of Pharmacy, The Methodist Hospital, 6565 Fannin Street, DB1-09 Houston, TX 77030, USA; ^2^J.C. Walter Jr. Transplant Center, The Methodist Hospital, 6550 Fannin Street, Smith Tower, Suite 1201, Houston, TX 77030, USA

## Abstract

The pharmacokinetics of tacrolimus are influenced by many factors, including genetic variability, acute infections, liver dysfunction, and interacting medications, which can result in elevated concentrations. The most appropriate management of acute tacrolimus toxicity has not been defined though case reports exist describing the therapeutic use of enzyme inducers to increase tacrolimus metabolism and decrease concentrations. We are reporting on the utilization of phenytoin to assist in decreasing tacrolimus concentrations in a case series of four solid organ transplant recipients with acute, symptomatic tacrolimus toxicity presenting with elevated serum creatinine, potassium, and tacrolimus trough concentrations greater than 30 ng/mL. All four patients had the potential causative agents stopped or temporarily held and were given 300 to 400 mg/day of phenytoin for two to three days. Within three days of beginning phenytoin, all four patients had a decrease in tacrolimus concentration to less than 15 ng/mL, a return to or near baseline creatinine concentration, and lack of phenytoin-related side effects. Therefore, phenytoin appears to be a safe and potentially beneficial treatment option in patients with symptomatic tacrolimus toxicity.

## 1. Introduction

In solid organ transplantation, tacrolimus (FK506) has emerged as the backbone of most immunosuppressive regimens [1]. Tacrolimus exerts its immunosuppressant effects by binding to the immunophilin FK506 binding protein (FKBP12), forming a complex which inhibits calcineurin-induced dephosphorylation of the transcription factor, nuclear factor of activated T cells (NFAT) [2]. This results in suppression of interleukin-2 (IL-2) transcription and inhibition of T-cell-mediated actions. Monitoring of tacrolimus concentrations is required due to its narrow therapeutic index, in order to maintain a balance between under-immunosuppression and subsequent rejection risk with over-immunosuppression and risk of toxicities.

Considerable variation exists in the pharmacokinetic profile of tacrolimus, resulting at times in challenges in maintaining therapeutic concentrations. Several factors affect the pharmacokinetics of tacrolimus, including the age or gender of the patient, liver impairment, and genetic variances in cytochrome P450 (CYP) enzymes and/or P-glycoprotein expression [3, 4]. Tacrolimus is extensively metabolized by the CYP3A4 isoenzyme, the most abundant of the CYP enzymes, constituting approximately one-third of the CYP enzymes found in intestinal lining and the liver [4]. It is also a substrate of P-glycoprotein (PGP) transport system. As a substrate of these systems, tacrolimus is subject to numerous drug-drug interactions. CYP3A4/PGP inhibitors may increase tacrolimus concentrations, resulting in potentially toxic concentrations and serious adverse effects such as neuro- or nephrotoxicity, whereas inducers may decrease tacrolimus concentrations resulting in suboptimal immunosuppression and an elevated risk for rejection.

Little data exists on the management of acute tacrolimus toxicities, although the use of enzyme inducers to enhance metabolism and lower concentrations has been described [5–9]. Phenytoin is a commonly used antiepileptic and a potent enzyme inducer. Herein, we report on the use of phenytoin to manage four cases of acute tacrolimus toxicity, themselves incited by drug interactions, requiring urgent reduction of tacrolimus concentrations.

## 2. Patient 1

A 53-year-old white male was admitted for acute kidney injury and hyperkalemia 21 months after liver transplantation for hepatitis C. He had a past medical history of human immunodeficiency virus (HIV), recurrent hepatitis C after transplant (grade 2, stage 2), and stage 3 chronic kidney disease (CKD). Approximately 2 weeks prior to admission, he was converted as an outpatient to a protease-inhibitor (atazanavir) based highly active antiretroviral therapy (HAART) regimen. He presented to clinic with nausea, vomiting, a serum creatinine (SCr) of 6.3 mg/dL (baseline 1.7 mg/dL), potassium of 6.7 mEq/L, and an FK trough level > 30 ng/mL. He was subsequently admitted for hydration, treatment of hyperkalemia, and telemetry monitoring. Despite sodium polystyrene administration, hydration, and withholding of tacrolimus, and HAART, on hospital day 2, SCr remained at 6.2 mg/dL, potassium was 6 mEq/L, and FK was >30 ng/mL. Due to the magnitude of renal impairment, lack of knowledge of the precise quantitative FK level, and unpredictability of tacrolimus clearance in the presence of atazanavir use, the decision was made to initiate phenytoin as a means to induce drug metabolism. Phenytoin was administered orally at a dose of 200 mg twice daily. FK concentration decreased to 22.3 ng/mL the day after phenytoin initiation and then to 11.7 ng/mL the next day. Phenytoin was continued for 3 days, and the patient was discharged 3 days after admission with a SCr of 3.9 mg/dL and potassium level within normal range. HAART therapy was reintroduced at discharge and at an outpatient visit 2 days later tacrolimus was reinitated. SCr eventually returned to near-baseline at 1 week after discharge, and has remained stable ever since.

## 3. Patient 2

A 57-year-old African American female had undergone heart transplantation secondary to nonischemic cardiomyopathy, with a retransplantation secondary to hyperacute rejection within 48 hours. Her postoperative course was further complicated by development of recurrent candida sternal wound infection requiring long-term antibiotic and antifungal therapy and intermittent wound VAC application. She was admitted 9 months after transplant with fevers, chest pain, and nausea two weeks after being discharged on fluconazole 400 mg by mouth daily. It was determined that the patient was taking an incorrect amount of tacrolimus as an outpatient, as she was admitted with a SCr of 7.3 mg/dL (baseline 1 mg/dL), potassium of 6.2 mEq/L, and FK trough level of >30 ng/mL. Despite reducing the fluconazole and holding tacrolimus on arrival, the SCr and FK levels remained at 7.4 mg/dL and >30 ng/mL, respectively, on the next day. Phenytoin was therefore initiated at a dose of 200 mg twice daily on the day after admission and continued for 2 days. In the subsequent days, FK level dropped from >30 to 22.3, 10.6, and 4.1, while SCr decreased to 2.6. Tacrolimus was reinitiated 6 days after admission, and SCr further declined to baseline by 10 days. Renal function remained stable for approximately 7 months after admission until the patient expired during an admission due to cardiac arrest.

## 4. Patient 3

A 70-year-old man underwent combined heart-kidney transplantation in November 2010 for end-stage renal disease and heart failure due to ischemic cardiomyopathy. He had a recent diagnosis of disseminated nocardia and was on stable treatment with sulfamethoxazole-trimethoprim and moxifloxacin. He presented one year after transplant with nausea, diarrhea, vomiting, a SCr of 4.2 mg/dL (baseline 0.8 mg/dL), potassium of 6.1 mEq/L, and FK trough level of >30 ng/mL. Unbeknownst to the transplant service the patient had also recently started taking vitamin/herbal supplements including a sustained release, high-potency multiple vitamin (Vit-Min 100, NOW Foods, Bloomingdale, IL, USA) as well as an omega-3 product (Super Omega-3, EPA/DHA with sesame lignans and olive fruit extract, Life Extension, Ft. Lauderdale, FL, USA). The FK level remained at >30 ng/mL and SCr at 4.5 mg/dL on the day after admission, at which point phenytoin was instituted orally at a dose of 100 mg three times daily. After 3 days of phenytoin, SCr and FK levels dropped to 1.5 mg/dL and 8.6 ng/mL, respectively. Tacrolimus was initiated 5 days after admission, and the patient's SCr returned to baseline 10 days after admission. 

## 5. Patient 4

A 58-year-old white male underwent deceased donor renal transplantation in January 2012 for end-stage renal disease secondary to hypertension and diabetes. He also had a history of diastolic heart failure, obesity, atrial fibrillation, and an early acute rejection episode treated with antithymocyte globulin. He was admitted to the intensive care unit nearly 6 months after transplant with fevers and progressive shortness of breath. The patient was subsequently found to have a pseudomonal pneumonia, as well as pulmonary nocardiosis. On admission, his SCr was 3.5 mg/dL (baseline of 2.1 mg/dL). In addition, potassium was 6.4 mEq/L, and FK trough level was 23.7. Empiric voriconazole was initiated on arrival prior to knowledge of the organisms or the FK level, and, despite holding tacrolimus, the concentration increased to >30 ng/mL with persistence of renal dysfunction on the day after arrival. In addition, the patient was experiencing new onset liver impairment with AST/ALT above 3 times the upper limit of normal and total bilirubin of 1.8 mg/dL. Given the circumstances, phenytoin was started at a dose of 200 mg by mouth twice daily and given for 3 days. After the last day of phenytoin administration, SCr and FK concentrations had declined to 1.8 mg/dL and 6.3 ng/mL, respectively. Tacrolimus was initated 6 days after admission, and SCr had improved to 1.5–1.7 mg/dL for the remainder of the admission and to the present day. 

## 6. Discussion

The pharmacokinetic profile of tacrolimus is well characterized [3]. Tacrolimus is metabolized almost entirely by the CYP3A enzymes found in the liver and intestinal wall. Expression of these enzymes can vary substantially between patients resulting in significant differences in metabolism. Additionally, a number of other factors can result in further changes in metabolism and drug concentrations including age, sex, comorbidities such as diabetes, hepatic impairment, hepatitis C and other acute infections, diet, and use of concomitant interacting medications [4]. Certain agents, including protease inhibitors, antifungal agents, and likely herbal supplements used by our patients, act as inhibitors of CYP3A resulting in a decrease in metabolism and an increase in bioavailability of tacrolimus. For example, the concomitant use of fluconazole with tacrolimus has been associated with a decrease in tacrolimus dose requirements by 40 to 60%, and empiric dosage decreases have been suggested by the manufacturer when used with other azole antifungals [2]. Additionally, tacrolimus dose requirements are decreased by 75 to 99% when administered with various protease inhibitors [10]. Various herbal supplements have been found to interact with tacrolimus and in general it is recommended that transplant recipients avoid them [11]. These interactions are experienced almost immediately upon starting the interacting agent as inhibition can occur as soon as the agent reaches the enzyme. When an enzyme inducing agent is used, the onset and length of induction can vary from days to weeks as these factors depend on the half-life of both the medication and CYP enzyme being induced [12, 13]. A report of 2 heart transplant patients demonstrated the variability of tacrolimus concentrations for up to 10 days after discontinuation of phenytoin [14]. Other studies have shown that catabolic activity of the CYP enzymes can be decreased during infection resulting in elevated drug levels [15, 16]. This suggests that elevated tacrolimus concentrations in our patients may have been in part due to acute infection as well.

Symptoms of acute tacrolimus toxicity vary widely, from lack of clinical symptoms to severe renal failure or neurotoxicity. Most often symptoms are mild and include nausea, headache, mild hand tremors, liver enzyme elevation, electrolyte disturbances, and mild increases in SCr [5, 6, 8, 17, 18]. No treatment recommendations exist for tacrolimus toxicity, as hemodialysis and plasma exchange are ineffective and other modalities such as gastric lavage and activated charcoal are only minimally effective and must be given early after administration [5, 19, 20]. In some reports, CYP3A4 inducers phenytoin and phenobarbital have been used in acute overdose settings to increase the clearance and facilitate lowering of the tacrolimus concentration [5–9]. These agents also have the additional benefit of seizure prevention as neurologic toxicities, including seizure and coma, are well documented with tacrolimus. Though rifampin is another strong CYP3A4 inducer known to decrease tacrolimus concentrations, we chose to use phenytoin for its antiepileptic properties [12, 21].

**Figure 1:**
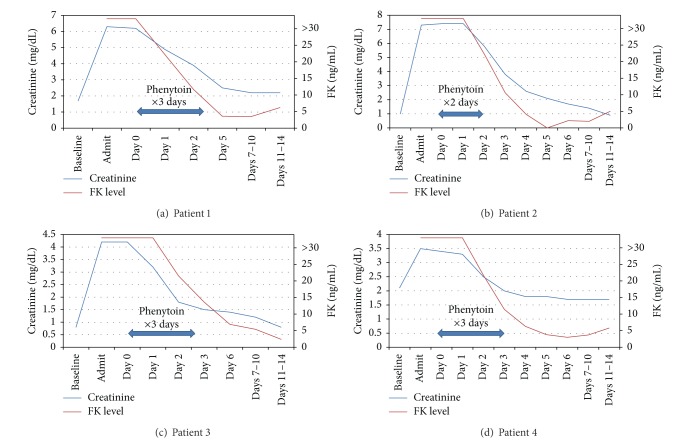
Mean creatinine and FK levels. Day 0 represents first day of phenytoin administration.

In our patients, symptoms were severe with SCr elevations from 67 to 630% of baseline and potassium levels of greater than 6 mEq/L (see Table 1). Due to the uncertainties in the metabolism and rate of clearance of tacrolimus under these circumstances and the severe degree of renal injury seen, we chose to administer phenytoin in our four patients. Phenytoin was chosen as it is hepatically metabolized and well tolerated at typical doses of 300 to 400 mg/day with the main side effects being gastrointestinal related. There are other common side effects that we did not expect to encounter as they are typically either dose related, such as the sedating effects on the central nervous system, or occur with long-term therapy, such as gingival hyperplasia. This interaction was utilized therapeutically in our patients with a successful decrease in tacrolimus levels to less than 15 ng/mL with 3 days of phenytoin, return of renal function to or near baseline in all four patients, and an absence of side effects (see Table 2 and Figure 1).

Our case series has some limitations. First, as our laboratory does not report specific tacrolimus concentrations greater than 30 ng/mL, the actual peak tacrolimus concentration was not accessible which makes it difficult to evaluate serial levels and describe the rate of elimination. Additionally, our case series did not include a comparator group that can be used to evaluate the difference in tacrolimus elimination and the actual effect of phenytoin on tacrolimus clearance. It is important to point out that oral phenytoin was utilized, rather than IV phenytoin, as our aim was also to induce the CYP3A enzymes in the GI tract as well as the liver resulting in further increases in tacrolimus elimination. IV phenytoin has also been associated with more side effects than the oral formulation, specifically hypotension during administration and injection site reactions.

In conclusion, short-term administration of the enzyme-inducer phenytoin facilitated reversal of elevated tacrolimus concentrations and severe renal impairment in four organ transplant recipients. Based on these results, phenytoin appears to be a potential treatment in severe cases of tacrolimus toxicity.

## Figures and Tables

**Table 1:** Summary of patients.

Patient number	Transplant type	Age race sex	Time since transplant (months)	Causative factor(s)	Clinical presentation	Baseline SCr (mg/dL)	Admit SCr (mg/dL)/% increase from baseline	Admit potassium (mEq/L)	FK level on admit (ng/mL)
1	Liver	53 W M	20.8	Atazanavir therapy, hepatitis C infection	Acute kidney injury, nausea, vomiting	1.7	6.3/270%	6.7	>30
2	Heart	57 AA F	9.2	Fluconazole therapy	Acute kidney injury, fever, chest pain	1	7.3/630%	6.2	>30
3	Heart/kidney	70 As M	12.4	Herbal supplements; disseminated Nocardia infection	Acute kidney injury, nausea, diarrhea	0.8	4.2/425%	6.1	>30
4	Kidney	58 W M	5.5	Voriconazole therapy, pseudomonas, and nocardia pneumonia	Acute kidney injury, fevers, shortness of breath	2.1	3.5/67%	6.4	23.7

**Table 2:** Management of FK toxicity and outcomes.

Patient number	Hospital day of phenytoin initiation	Phenytoin dose (mg/day)/duration (days)	Other management	Days to FK <15 ng/mL	Days to resuming tacrolimus	Days to baseline SCr	Outcome/followup (months)
1	1	400/3	HAART temporarily withheld	2	5	∗	Alive, stable renal function/60
2	1	400/2	Fluconazole temporarily withheld	3	5	10	Died, cardiac arrest/7
3	1	300/3	Herbal products stopped	3	4	10	Alive, stable renal function/13
4	2	400/3	Voriconazole stopped	3	4	3	Alive, stable renal function/8

*Patient baseline creatinine 1.7 mg/dL did not return back to baseline but settled with creatinine of 2.2 mg/dL.
